# Immunolocalization of calcium sensing and transport proteins in the murine endolymphatic sac indicates calciostatic functions within the inner ear

**DOI:** 10.1007/s00441-019-03062-2

**Published:** 2019-07-23

**Authors:** David Bächinger, Hannes Egli, Madeline M. Goosmann, Arianne Monge Naldi, Andreas H. Eckhard

**Affiliations:** 1 Department of Otorhinolaryngology, Head and Neck Surgery, University Hospital Zurich, Frauenklinikstrasse 24, 8091 Zurich, Switzerland; 2 University of Zurich, Zurich, Switzerland

**Keywords:** Endolymphatic hydrops, Inner ear, Meniere’s disease, Mitochondria-rich cells, NCX2, SERCA1, SERCA2, TRPV5, TRPV6, PMCA1, PMCA4, Ribosome-rich cells

## Abstract

An exceptionally low calcium (Ca^2+^) concentration in the inner ear endolymph ([Ca^2+^]_endolymph_) is crucial for proper auditory and vestibular function. The endolymphatic sac (ES) is believed to critically contribute to the maintenance of this low [Ca^2+^]_endolymph_. Here, we investigated the immunohistochemical localization of proteins that are presumably involved in the sensing and transport of extracellular Ca^2+^ in the murine ES epithelium. Light microscopic and fluorescence immunolabeling in paraffin-embedded murine ES tissue sections (male C57BL/6 mice, 6–8 weeks old) demonstrated the presence of the calcium-sensing receptor CaSR, transient receptor potential cation channel subtypes TRPV5 and TRPV6, sarco/endoplasmic reticulum Ca^2+^-ATPases SERCA1 and SERCA2, Na^+^/Ca^2+^ exchanger NCX2, and plasma membrane Ca^2+^ ATPases PMCA1 and PMCA4 in ES epithelial cells. These proteins exhibited (i) membranous (apical or basolateral) or cytoplasmic localization patterns, (ii) a proximal-to-distal labeling gradient within the ES, and (iii) different distribution patterns among ES epithelial cell types (mitochondria-rich cells (MRCs) and ribosome-rich cells (RRCs)). Notably, in the inner ear membranous labyrinth, CaSR was exclusively localized in MRCs, suggesting a unique role of the ES epithelium in CaSR-mediated sensing and control of [Ca^2+^]_endolymph_. Structural loss of the distal ES, which is consistently observed in Meniere’s disease, may therefore critically disturb [Ca^2+^]_endolymph_ and contribute to the pathogenesis of Meniere’s disease.

**Electronic supplementary material:**

The online version of this article (10.1007/s00441-019-03062-2) contains supplementary material, which is available to authorized users.

## Introduction

Hair cell function in the cochlea and the vestibular system is calcium (Ca^2+^)-dependent (Tanaka et al. 1980; Ohmori 1985; Kozel et al. 1998) and requires the endolymphatic Ca^2+^ concentration ([Ca^2+^]_endolymph_) to be tightly controlled at a level that is unusually low (0.017–0.133 mmol/l (Salt et al. 1989)) for an extracellular fluid (e.g., [Ca^2+^]_blood plasma_ = 1.2 mmol/l (Diem and Lentner 1970)). To maintain this physiological [Ca^2+^]_endolymph_, numerous epithelial sites within the membranous labyrinth are engaged in Ca^2+^ transport across the endolymph-perilymph barrier, utilizing Ca^2+^-permeable channels (e.g., transient receptor potential cation channel subfamily V members [TRPV channels] (Takumida et al. 2005; Wangemann et al. 2007; Nakaya et al. 2007; Ishibashi et al. 2008)), ion exchangers (e.g., sodium-calcium exchangers [NCX] (Oshima et al. 1997; Yamauchi et al. 2010)), and Ca^2+^ ATPases (e.g., plasma membrane calcium ATPase [PMCA] (Crouch and Schulte 1995; Ågrup et al. 1999)). Many of these transport proteins are directly regulated downstream targets of the calcium sensing receptor (CaSR), a G-coupled cell surface receptor that is prominently expressed in the primary tissues involved in whole-body Ca^2+^ homeostasis, including the kidney and the parathyroid and thyroid glands (Blankenship et al. 2001; Topala et al. 2009; Ranieri et al. 2013). In those tissues, CaSR senses changes in Ca^2+^ levels in urine and plasma and modulates the activity and expression of Ca^2+^ transport proteins to increase renal Ca^2+^ reabsorption (Riccardi et al. 1998) and the Ca^2+^-mediated release of calciotropic hormones (parathyroid hormone, calcitonin) (Brown and MacLeod 2001), respectively. In the inner ear, the endolymphatic sac (ES), among other epithelial tissues, is a site of CaSR gene expression (Beitz et al. 1999; Lin et al. 2018) and critically contributes to endolymphatic Ca^2+^ homeostasis. This function was first indicated by in vivo animal experiments in which surgical separation of the ES resulted in a nonphysiological increase in [Ca^2+^]_endolymph_ and endolymphatic hydrops development in the cochlea (Ninoyu and Meyer zum Gottesberge 1986; Salt and DeMott 1994). Structural loss of the distal (extraosseous) ES, as induced in this animal model, is a histopathological hallmark and a presumed etiopathogenic factor in Meniere’s disease (MD) (Eckhard et al. 2019b).

Here, we investigated the immunolocalization patterns of CaSR and Ca^2+^ transport proteins in the mature murine ES epithelium to elucidate the molecular determinants of Ca^2+^ homeostasis in the ES and to better understand the pathophysiological consequences of the structural loss of the ES in MD.

## Materials and methods

### Animals

All animal experiments were performed according to Swiss animal welfare laws and were approved by the local veterinary authorities (Kantonales Veterinäramt Zürich; protocol no. ZH269/16). Male, 6- to 8-week-old C57BL/J6-Crl1 mice were purchased from Charles River Laboratories, Inc. (Sulzfeld, Germany). A total of 12 animals were used in this study. All animals were kept at an in-house animal facility with free access to a standard chow diet and water under a standard 12 h light/dark cycle.

### Tissue preparation, fixation, decalcification, embedding, and sectioning

Animals were sacrificed by CO_2_ inhalation. For tissue fixation, the thoracic cavity was opened, and a cannula was inserted in the left cardiac ventricle for transcardial perfusion with 10 ml phosphate buffered saline (PBS), followed by 10 ml of a fixative, i.e., (i) 10% neutral buffered formalin (F; Lucerna-Chem AG, Luzern, Switzerland), (ii) F with 1% acetic acid (FA; Sigma-Aldrich, Steinheim, Germany), (iii) F with 0.2% glutaraldehyde (GA; Sigma-Aldrich), or (iv) FA with 0.2% glutaraldehyde (FGA). The respective fixative used in each protocol is given in Table 1. Then, the animals were decapitated, soft tissues were removed from the outer surface of the skull using a sharp scalpel, and the cranial part of the skull was opened to remove most of the cerebrum, while the cerebellum was left in situ. The tympanic bullae were opened to allow faster penetration of the fixative into the inner ear. The specimens were then immersed in the respective fixative used for transcardial perfusion for 6 h on a countertop shaker. Afterwards, the specimens were dehydrated in a graded series of ethanol solutions (50%, 70%, 95%, and 100%), embedded in paraffin and sectioned at 4 μm using an HM 355S Automatic Microtome (Thermo Fisher Scientific, Waltham, MA, USA). Sections were collected on SuperFrost Plus slides (Thermo Fisher Scientific), dried on a heating plate at 37 °C overnight and stored at room temperature.

**Table 1:** Primary antibodies used with DAB labeling. Dilutions indicate the highest dilution producing a sufficient signal. All antibodies were diluted in 1% normal horse serum (NHS). Form indicates 10% neutral buffered formalin. *FA* form + 1% acetic acid, *FG* form + 0.2% glutaraldehyde, *FGA* FA + 0.2% glutaraldehyde, *HIER* heat-induced epitope retrieval

Epitope	Calbindin D-28 k	CaSR	NCX2	Parvalbumin	pPMCA	PMCA1	PMCA2	SERCA1	SERCA2	TRPV5	TRPV6
Immunogen (peptide sequence)	No info	ADDYGRPGIEKFREEAEE RDI	RVGDAQGMFEPDGG	No info	Exact sequence not available^a^	SGVKNSLKEANHD	KEIPDPSSINAKTLE	TPDQVKRHLEKYG	CTPNKPSRTSMSK	GLNLSEGDGEEVYHF	NRGLEDGESWEYQI
Fixative	FG	FA	FG	FA	Form, FG	FG	FG	FA	FA	Form, FG	Form, FG
HIER	No	Yes	Yes	Yes	No	No	No	No	No	Yes	Yes
Primary antibody	Monoclonal anti-calbindin D-28 k (mouse)	Monoclonal anti-CaSR (mouse)	Polyclonal anti-NCX-2 (rabbit)	Monoclonal antiparvalbumin (mouse)	Monoclonal anti-pPMCA (mouse)	Polyclonal anti-PMCA1 (rabbit)	Polyclonal anti-PMCA2 (rabbit)	Polyclonal anti-SERCA1 (rabbit)	Polyclonal anti-SERCA2 (rabbit)	Polyclonal anti-TRPV5 (rabbit)	Polyclonal anti-TRPV6 (rabbit)
Dilution	1:500	1:15′000	1:500	1:500	1:4000	1:1000	1:1000	1:500	1:500	1:200	1:200
Manufacturer	Swant, Martiny, Switzerland	Novus Biologicals, Minneapolis, USA	Alomone Labs, Jerusalem, Israel	Swant, Martiny, Switzerland	Abcam, Cambridge, UK	Alomone Labs, Jerusalem, Israel	Alomone Labs, Jerusalem, Israel	Alomone Labs, Jerusalem, Israel	Alomone Labs, Jerusalem, Israel	Alomone Labs, Jerusalem, Israel	Alomone Labs, Jerusalem, Israel
Catalog number	300	NB120-19347SS	ANX-012	235	ab2825	ACP-005	ACP-002	ACP-011	ACP-012	ACC-035	ACC-036
Secondary antibody (biotinylated)	Anti-mouse IgG (donkey)	Anti-mouse IgG (donkey)	Anti-rabbit IgG (donkey)	Anti-mouse IgG (donkey)	Anti-mouse IgG (donkey)	Anti-rabbit IgG (donkey)	Anti-rabbit IgG (donkey)	Anti-rabbit IgG (donkey)	Anti-rabbit IgG (donkey)	Anti-rabbit IgG (donkey)	Anti-rabbit IgG (donkey)
Dilution	1:400	1:400	1:400	1:400	1:400	1:400	1:400	1:400	1:400	1:1000	1:1000
Manufacturer	Milan Analytica AG, Rheinfelden,	Milan Analytica AG	Milan Analytica AG	Milan Analytica AG	Milan Analytica AG	Milan Analytica AG	Milan Analytica AG	Milan Analytica AG	Milan Analytica AG	Milan Analytica AG	Milan Analytica AG
Catalog number	715-065-151	715-065-151	715-065-152	715-065-151	715-065-151	715-065-152	715-065-152	715-065-152	715-065-152	715-065-152	715-065-152

^a^This antibody recognizes an epitope between amino acids 724–783 of the human erythrocyte calcium pump

### Immunohistochemical DAB labeling

Sections were deparaffinized in Histo-Clear (National Diagnostics, Atlanta, GA, USA), rehydrated in a graded series of ethanol solutions (100%, 95%, 70%, and 50%), and rinsed in tap H_2_O. When required, heat-induced antigen retrieval (HIAR) with pressurized coverslipping of the mounted tissue sections was performed (Table 1) according to a previously established protocol (Eckhard et al. 2019a). All subsequent steps were performed at room temperature. Nonspecific binding was blocked with 1% normal horse serum (NHS) for 15 min, followed by incubation with primary antibodies overnight. The sections were then incubated with biotinylated secondary antibodies for 1 h, followed by incubation with avidin-biotin-HRP complex (Vectastain ABC HRP Kit, Vector Laboratories, Burlingame, CA, USA). Visualization was performed with 3,3′-diaminobenzidine (DAB) (DAB Peroxidase [HRP] Substrate Kit, Vector Laboratories). All incubation steps were performed at room temperature, and each incubation step was followed by rinsing the sections in PBS for 5 min. All primary and secondary antibody combinations utilized in this study are listed in Table 1. Negative controls for all immunolabeling experiments were obtained by omitting the primary antibody or after preabsorption of the primary antibodies with the corresponding commercially available control peptides (for primary antibodies against TRPV5/6, PMCA1/4, NCX2, and SERCA1/2). For nuclear counterstaining, the slices were incubated for 5 s with hematoxylin diluted in distilled water (1:6) and mounted with a permanent mounting medium (VectaMount, Vector Laboratories).

### Immunofluorescence double labeling

Deparaffinization, rehydration of the sections, and incubation with primary antibodies was performed as described for DAB-labeling experiments. All primary antibodies used for immunofluorescence double labeling are listed in Table 2. The sections were then incubated with biotinylated antibodies raised in donkey and directed against mouse (1:400; 715-065-151, Milan Analytica AG, Rheinfelden, Switzerland) and Alexa Fluor 594-conjugated antibodies raised in goat and directed against rabbit (1:400; 111-585-144, Milan Analytica AG) for 1 h, followed by incubation with avidin-biotin-HRP complex (Vectastain ABC HRP Kit, Vector Laboratories). Biotinylated tyramine (10 min) and a subsequent second incubation with avidin-biotin-HRP complex were used for signal amplification. Visualization was performed with Alexa Fluor 488-conjugated streptavidin (1:800; Milan Analytica AG) and Alexa Fluor 594-conjugated anti-goat antibodies raised in donkey (1:400; 705-585-003, Milan Analytica AG). The sections were mounted with an aqueous mounting medium containing DAPI for nuclear counterstaining (H-1200, Vectashield, Vector Laboratories).

**Table 2:** Primary antibodies used with fluorescence labeling. Dilutions indicate the highest dilution producing a signal. All antibodies were diluted in 1% normal horse serum (NHS). If not otherwise specified, sodium citrate (10 mM) at pH 6.0 was used as the HIER buffer. Form indicates 10% neutral buffered formalin. *FA* form + 1% acetic acid, *FG* form + 0.2% glutaraldehyde, *FGA* FA + 0.2% glutaraldehyde, *HIER* heat-induced epitope retrieval, *NHS* normal horse serum

Epitope	CaSR	γENaC	TRPV5	V-ATPase
Immunogen (peptide sequence)	ADDDYGRPGIEKFREEAEERDI	Available upon request.	GLNLSEGDGEEVYHF	MAMEIDSRPGGLPGSSCNLGAAREHMQAVTRNYITHPRVTYRTVCSVNGPLVVLDR VKFAQYAEIV
Fixative	Form	Form	FG	FA
HIER	Yes	Yes	Yes	Yes (Tris-EDTA solution, 10 mM / 1 mM, at pH 9.0)
Primary antibody	Monoclonal anti-CaSR (mouse)	Monoclonal anti-γENaC (rabbit)	Polyclonal anti-TRPV5 (rabbit)	Polyclonal anti-Vacuolar-type H^+^ ATPase subunit B, kidney isoform (rabbit)
Dilution	1:200	1:200	1:100	1:200
Manufacturer	Novus Biologicals, Minneapolis, USA	Gift from Prof. Johannes Loffing, University of Zurich, Switzerland	Alomone Labs, Jerusalem, Israel	Novus Biologicals
Catalog number	NB120-19347SS	n. a.	ACC-035	NBP2-33962

### Microscopy analysis

Images were acquired using either a Leica DMI6000 microscope (Leica, Wetzlar, Germany) or a Leica SP5 confocal laser scanning microscope (Leica) and processed with Adobe Photoshop CS5 software (Version 12.0, Adobe Systems, San Jose, CA, USA).

### Quantification of immunolabeled ES epithelial cells

DAB-labeled (DAB^+^) ES epithelial cells were quantified on light microscopic (× 40 air objective) images of hematoxylin-counterstained tissue sections. Cell counting was performed in at least three non-consecutive sections from two different axial planes of the intraosseous ES (iES) and the extraosseous ES (eES). Each section was divided into ten equally long segments along the longitudinal axis of the ES. The percentage of DAB^+^ cells among the epithelial cells with hematoxylin-stained nuclei was determined in each segment.

Single- and double-immunofluorescence-labeled ES epithelial cells in the eES were quantified following double immunofluorescence labeling of CaSR with either V-ATPase, TRPV5, or γENaC. The percentages of fluorescence-labeled cells among the epithelial cells with DAPI-counterstained nuclei were determined. From each double immunofluorescence labeling experiment, at least 100 labeled cells were counted, and sections from at least 3 independent specimens were analyzed.

### Descriptive statistics

Statistical analyses were performed using Prism software (version 7.0, GraphPad Software). Cell counts are expressed as percentages of the number of epithelial cells in a segment of the ES. Mean values and standard deviations derived from six animals are shown.

## Results

Based on the previously demonstrated similarities between the ES epithelium and the kidney distal tubular epithelium on the cellular and molecular levels, we mainly focused our immunohistochemical analysis on Ca^2+^ transport-associated proteins that are known to be crucial for the renal tubular handling of calcium.

### Cellular immunolocalization patterns of the CaSR and Ca^2+^ transport proteins

**Fig. 1:**
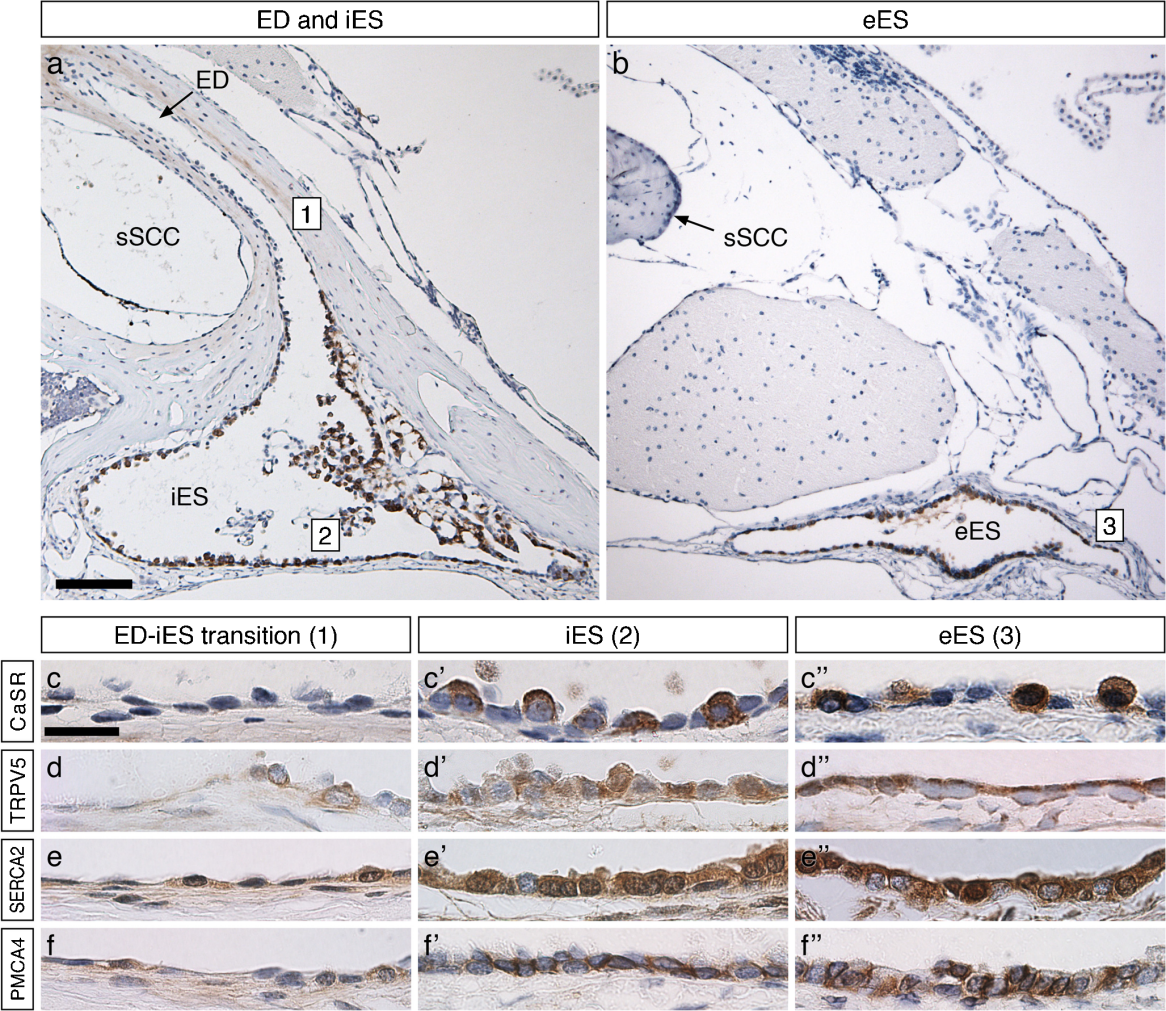
DAB-immunohistochemical staining of the calcium sensing receptor (CaSR) and selected calcium transport proteins along the murine endolymphatic sac. **a**, **b** Overviews of the iES emerging from the ED (**a**) and of the eES (**b**), demonstrating epithelial CaSR immunostaining along the ES. Boxed numbers indicate representative regions shown below in columns. **c**–**f** Representative images corresponding to the regions indicated by the boxed numbers in (**a**) and (**b**), i.e., the ED-iES transition (first column), iES (second column), and eES (third column). Scale bars: 100 μm (**a**), 20 μm (**c**). ED endolymphatic duct, eES extraosseous endolymphatic sac, iES intraosseous endolymphatic sac, sSCC superior semicircular canal

DAB immunolabeling of the CaSR, as well as of TRPV5, TRPV6, SERCA1, SERCA2, NCX2, PMCA1, and PMCA4 was visible in the iES (proximal to the operculum) and eES (distal to the operculum; Fig. 1 and Supplementary Fig. 1) portions of the ES epithelium. Staining of proteins was absent or very weak in the endolymphatic duct (ED; Fig. 1 and Supplementary Fig. 1). Negative controls (omitted primary antibodies and preabsorption of primary antibodies with control peptides (TRPV5/6, NCX2, PMCA1/4, SERCA1/2) showed no specific staining in the murine ES epithelium.

CaSR immunolabeling was present in the iES and eES portions (Fig. 1a, b) and exhibited a scattered labeling pattern within the epithelium. In CaSR-labeled cells, strong cytoplasmic and membranous labeling was found, which in many cells was polarized to the basolateral membranes (Fig. 1c–c”). Immunolabeling signals for transient receptor potential cation channel subfamily V (TRPV) members  TRPV5 and TRPV6 appeared to be cytoplasmic with strong polarization towards the apical cell pole, most clearly in epithelial cells in the eES (Fig. 1d–d” and Supplementary Fig. 1A-A”). Homogeneous cytoplasmic immunolabeling of the sarcoplasmic/endoplasmic reticulum calcium ATPase (SERCA) subtypes SERCA1 and SERCA2 was found throughout the iES and eES epithelium (Fig. 1e–e” and Supplementary Fig. 1B-B”) and was consistent with their known localization in the endoplasmic reticulum (Strehler and Treiman 2004). Labeling for the plasma membrane calcium ATPase (PMCA) subtypes PMCA1 and PMCA4, as well as for the sodium-calcium exchanger NCX2, was polarized in the basolateral membranes, most clearly in the eES (Fig. 1f–f” and Supplementary Fig. 1). No immunolabeling in the ES and ED was found for PMCA2 and NCX1, nor for the Ca^2+^-binding proteins Calbindin D-28k and parvalbumin (Supplementary Fig. 2). All immunolabeling patterns and their cellular polarization in the ES epithelium were consistent with those previously described for the distal convoluted tubule and the connecting tubule epithelium (Loffing et al. 2001; Loffing and Kaissling 2003); positive control experiments are shown in Supplementary Fig. 3).

### Spatial (proximal-to-distal) immunolabeling gradients

**Fig. 2:**
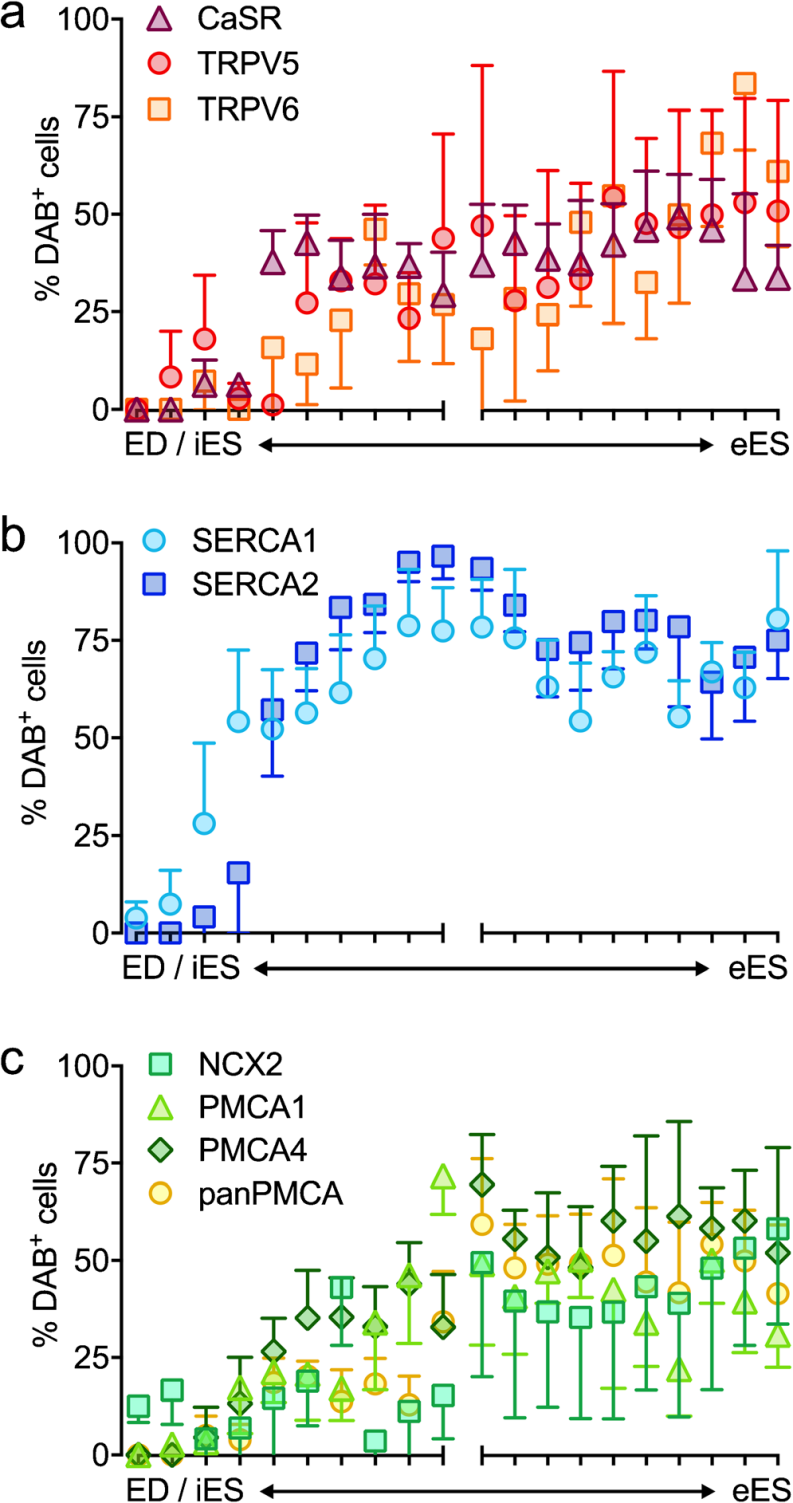
Quantification of DAB^+^ cells along the ES reveals staining gradients along the ES epithelium increasing from the iES to the eES. The *y*-axis shows the percentage of DAB^+^ cells relative to all ES epithelial cells. For cell counting, both the iES and eES were each divided into ten equal segments. The ticks on the *x*-axis correspond to each of the ten consecutive segments of the iES (left *x*-axis) and eES (right *x*-axis). The first two segments represent the endolymphatic duct epithelium. The values are the percentages of the total number of cells in the respective ES segment and are given as mean values ± SD from six animals. **a** Quantification of DAB^+^ cells for CaSR and the apical Ca^2+^ channels TRPV5 and TRPV6. **b** Quantification of DAB^+^ cells for the cytoplasmatic proteins SERCA1 and SERCA2. **c** Quantification of DAB^+^ cells for proteins located in the basolateral membrane

For each protein studied, DAB-labeled cells were quantified along the longitudinal (proximal-to-distal) axis of the ES epithelium, reaching from the proximal transition of the iES in the ED to the distal blind end of the eES. For all of the investigated proteins, a gradual increase in the immunolabeling signal in the proximal-to-distal direction was observed. At the very distal end of the eES portion, on average, more than 50% of the epithelial cells were positively labeled for most of the investigated proteins (Fig. 2).

### Differential immunolocalization patterns in mitochondria-rich and ribosome-rich cells

**Fig. 3:**
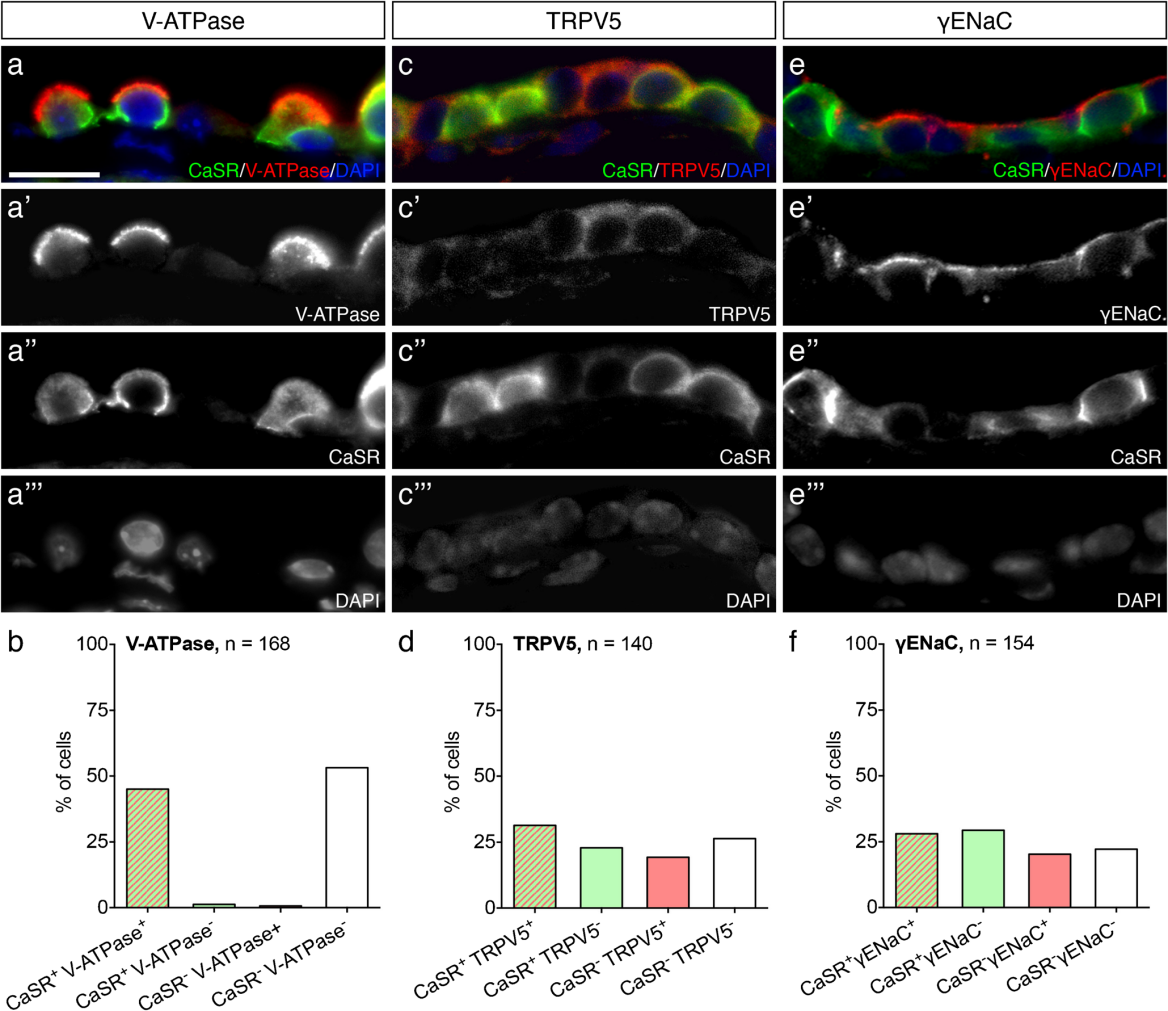
Quantitative colocalization of CaSR with V-ATPase, TRPV5, and γENaC in the eES. Representative images and quantification of fluorescent immunohistochemical double labeling of CaSR and V-ATPase (**a**–**a”’**, **b**), TRPV5 (**c**–**c”’**, **d**), and γENaC (**e**–**e”’**, **f**). Scale bar: 10 μm. *N* total number of cells counted

We found scattered DAB-immunolabeling patterns within the ES epithelium for almost all of the investigated proteins (Fig. 1). We therefore investigated whether CaSR and Ca^2+^ transport proteins are differentially distributed among the two major ES epithelial cell types, i.e., mitochondria-rich cells (MRCs) and ribosome-rich cells (RRCs) (Lundquist et al. 1964; Barbara et al. 1987; Dahlmann and von Düring 1995), using immunofluorescence double labeling. Labeling of the MRC-specific vacuolar-type H^+^-ATPase (V-ATPase; (Stanković et al. 1997; Dou et al. 2004)) and CaSR in the eES portion showed a strict cellular colocalization of both proteins (Fig. 3a–a''') in 45.0% of the cells (Fig. 3b), indicating the localization of CaSR in MRCs (vATPase^+^/CaSR^+^) but not in RRCs (vATPase^−^/CaSR^−^). Colabeling of TRPV5 and CaSR (Fig. 3c-c''', d) was found in 31.4% of the cells (TRPV5^+^/CaSR^+^), while the other cells showed exclusive labeling for either TRPV5 (TRPV5^+^/CaSR^−^, 19.3%) or CaSR (TRPV5^−^/CaSR^+^, 22.9%) or were devoid of labeling (TRPV5^−^/CaSR^−^, 26.4%). Colabeling of the gamma subunit of the epithelial sodium channel (γENaC) and CaSR (Fig. 3e-e''', f) was present in 28.1% of the cells (γENaC^+^/CaSR^+^), while 20.3% of cells showed labeling for only γENaC (γENaC^+^/CaSR^−^), 29.4% of the cells were labeled for only CaSR (γENaC^−^/CaSR^+^), and 22.2% of cells were devoid of labeling (γENaC^−^/CaSR^−^).

### CaSR immunolabeling within the murine inner ear is restricted to the ES epithelium

**Fig. 4:**
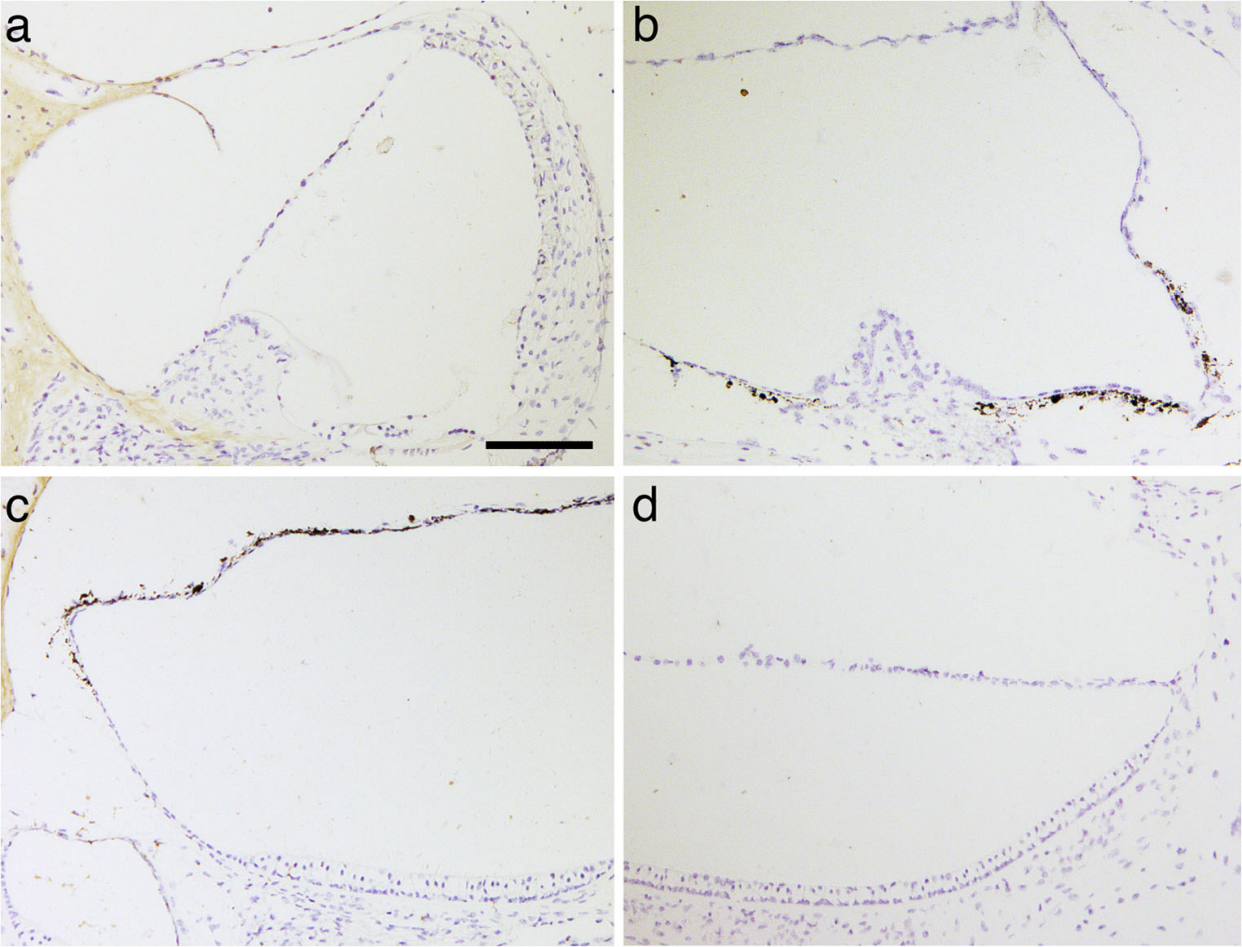
No CaSR immunolabeling was detected within the cochlea or the vestibular end organs. **a** Negative CaSR immunolabeling in the cochlea, in particular the stria vascularis, spiral ligament, Reissner’s membrane, limbus, organ of Corti, and spiral ganglion. **b** Ampullary region of the lateral semicircular canal. No CaSR staining was observed in the crista or the SCC epithelium (membranous labyrinth). Adjacent to the crista, several dark cells (melanocytes) are interspersed in the membranous labyrinth. **c**, **d** No CaSR immunolabeling was found in the sensory epithelium or surrounding tissue of the utricle (**c**) and saccule (**d**). Several dark cells are interspersed in the membranous labyrinth covering the utricle (**c**)

No specific CaSR immunoreactivity was found in other portions of the membranous labyrinth, including the cochlea, saccule, utricle, and semicircular canal cristae (Fig. 4).

## Discussion

In the present study, the ES epithelium was identified as an exclusive site of CaSR immunolocalization within the mature murine inner ear. Several CaSR-regulated Ca^2+^ transport proteins (TRPV5, PMCA1/4), as well as non-CaSR-regulated Ca^2+^ channels/transporters (TRPV6, NCX2), and intracellular Ca^2+^ sequestering proteins (SERCA1/2) were localized in the ES epithelium, almost all of which exhibited their highest immunolabeling intensities in the distal eES portion.

Consistent with the immunolocalization of CaSR in MRCs of the mature murine ES shown here, previous gene expression studies detected CaSR mRNA expression in MRCs of the early postnatal murine ES (Honda et al. 2017), in the adult rat ES (Beitz et al. 1999), and, moreover, in the adult rat organ of Corti, stria vascularis, Reissner’s membrane, and vestibulum (Beitz et al. 1999), as well as in neuromast hair cell stereocilia of zebrafish larvae (Lin et al. 2018). The results of the present study indicate that the CaSR protein in the mature murine inner ear is exclusively expressed in the ES or that immunolabeling at other cellular sites was below the detection level. We propose that the CaSR may have a unique role in sensing and regulating [Ca^2+^]_endolymph_ in the ES, with significance for the overall endolymphatic Ca^2+^ homeostasis in the inner ear. However, it is unknown whether the CaSR enables ES epithelial cells to sense Ca^2+^ changes in the ES endolymph, the cerebrospinal fluid, or in both extracellular fluid compartments that face the ES epithelium. Moreover, the molecular signaling cascades by which the CaSR may regulate downstream effector proteins, such as TRPV5 and PMCA1/4 (Blankenship et al. 2001; VanHouten et al. 2007; Topala et al. 2009), which are also localized in the ES epithelium (Ågrup et al. 1999; Honda et al. 2017; present study), require further exploration. Although CaSR function in the ES may contribute to maintaining Ca^2+^ within its narrow physiological range in the entire inner ear, its effects on [Ca^2+^]_endolymph_ in the vestibular and cochlear portions are probably slow and may have physiological relevance primarily on longer time scales. This assumption is supported by previous observations that (i) longitudinal fluid movements (between endolymph compartments) virtually do not occur, at least under physiological conditions (Salt et al. 1986), and that (ii) surgical separation of the ES only leads to a slow rise of [Ca^2+^]_endolymph_ over a time course of several weeks (Ninoyu and Meyer zum Gottesberge 1986; Salt and DeMott 1994). This may be primarily due to local ion homeostatic mechanisms within the cochlea and the vestibular portions (Lundquist 1976; reviewed in Salt 2010), as well as anatomical diffusion barriers, such as the utriculosaccular duct, which (in the adult murine inner ear) largely prevents fluid exchange between the pars inferior (cochlea, saccule) and the pars superior (utricle, semicircular canals) (Cantos et al. 2000). In addition, the CaSR may have local homeostatic functions in the ES, such as maintaining ES epithelial integrity or mediating immune responses (Cheng et al. 2014).

Among the two main epithelial cell types in the ES, i.e., MRCs and RRCs, we found that the CaSR was exclusively localized in vATPase^+^ MRCs (Dou et al. 2004; Honda et al. 2017). This cell-type-specific distribution of CaSR resembles that in intercalated cells in the renal cortical collecting duct (CCD; (Everett 2006)). On the subcellular level, CaSR was polarized towards both membrane domains (apical and basolateral) with additional strong cytoplasmic labeling. This finding is consistent with the distribution of CaSR in the distal renal tubule epithelium (distal convoluted tubule and collecting duct (Riccardi et al. 1998)), where CaSR regulates transepithelial Ca^2+^ reabsorption from the tubular fluid via TRPV5 (Topala et al. 2009) and PMCA (Blankenship et al. 2001). Notably, other shared molecular mechanisms of transepithelial ion (Na^+^) transport between the ES epithelium and the distal renal tubule epithelium have been reported (Mori et al. 2017; Eckhard et al. 2019b). In contrast to CaSR, TRPV5 was differentially localized in subsets of MRCs and RRCs, thereby differing from its exclusive localization in principal cells in the renal CCD (Loffing and Kaissling 2003). However, this non-cell-type-specific TRPV5 localization in the ES is consistent with the RNA sequencing analysis of the murine ES (Honda et al. 2017). These and previously noted structural differences between the epithelia of the ES and the renal CCD (Wangemann and Marcus 2017), despite their numerous shared cellular and molecular features, may be due to the different physiological requirements for maintaining extracellular Ca^2+^ homeostasis in the ES endolymph and the blood plasma. In addition to CaSR-regulated Ca^2+^ transporters, we identified TRPV6 and NCX2 in the ES epithelium, both of which are known to enable constitutively active Ca^2+^ transport in the renal tubular epithelium (Loffing and Kaissling 2003).

Loss of the proposed Ca^2+^-homeostatic function of the ES may be of significance in various pathological conditions, such as otoconial disorders and MD. The biogenesis of otoconial Ca^2+^ carbonate (CaCO_3_) crystals is believed to occur in the ES luminal microenvironment and is sensitive to changes in [Ca^2+^]_endolymph_ (Nakaya et al. 2007). For example, disturbed formation of otoconia was observed in Foxi1 knockout (k.o.) mice and Efnb2 k.o. mice (Hulander 2003; Raft et al. 2014). In these models, the MRC population in the ES is developmentally lacking (Hulander 2003) or mildly decreased and proximally mislocalized in the ES (Raft et al. 2014), respectively. The loss of CaSR-regulated Ca^2+^ homeostasis in the ES is one possible explanation for the compromised otoconia formation in these transgenic mouse models. Another explanation is the loss of (MRC-specific) pendrin- and v-ATPase-mediated anion transport that leads to disturbances of endolymphatic pH, which in turn may inhibit the pH-sensitive TRPV5/6 channels in the ES epithelium and thereby disturb [Ca^2+^]_endolymph_ ((Nakaya et al. 2007), present study). In MD, the structural loss of the distal ES epithelium is a consistent histopathological hallmark. Presuming that the human ES harbors Ca^2+^ transport mechanisms in proximal-to-distal expression gradients analogous to the murine ES, structural loss of the distal ES—a pathological hallmark in MD (Eckhard et al. 2019b)—and in particular its Ca^2+^ transport functions may be crucial in the pathogenesis of endolymphatic hydrops (Ninoyu and Meyer zum Gottesberge 1986; Salt and DeMott 1994).

In conclusion, the murine distal ES epithelium presumably has a “calciostatic” function for inner ear fluid homeostasis. Human pathology of the ES likely impairs Ca^2+^ homeostasis of the inner ear fluids and is therefore potentially a significant pathophysiological factor in various inner ear disorders.

## Electronic supplementary material

Figure S1 DAB-immunohistochemical staining of selected calcium transport proteins along the murine endolymphatic sac. (**A – E**) Representative images corresponding to the regions indicated by the boxed numbers in Fig. 1a and b, i.e., the ED-iES transition (first column), iES (second column) and eES (third column). Scale bar: 20 μm. (PNG 165460 kb)

High resolution image (TIFF 3415 kb)

Figure S2 A lack of DAB-immunohistochemical staining in the ES epithelium was observed for calbindin-D28k, parvalbumin, PMCA2 and NCX1. (**A**) Negative DAB immunolabeling of calbindin-D28k in the ES epithelium. Scale bar (for A, D, G and I): 20 μm. (**B – C**) Positive control DAB staining for calbindin-D28k in the distal nephron (B) and in Purkinje cells of the cerebellar cortex (C). Scale bar in (B): 10 μm (for B, E and J); scale bar in (C): 10 μm (for C, F and H). (**D**) Negative DAB immunolabeling of parvalbumin in the ES epithelium. (**E – F**) Strong positive control DAB staining in parvalbumin-immunostained cells in the distal nephron (E) and weak positive DAB staining in Purkinje cells of the cerebellar cortex (F). (**G**) Negative DAB immunolabeling of PMCA2 in the ES epithelium. (**H**) Positive control staining of PMCA2, with a predominant apical staining pattern in Purkinje cells of the cerebellar cortex (F). (**I**) Negative DAB immunolabeling of NCX1 in the ES epithelium. (**J**) Positive control staining for NCX1, with a basolateral staining pattern in the distal nephron (J). All scale bars: 20 μm. (PNG 91011 kb)

High resolution image (TIFF 1899 kb)

Figure S3 Positive DAB control staining in the kidney and in the trigeminal ganglion for proteins exhibiting positive immunostaining in the ES epithelium (Fig. 1, Fig. S1). (**A – H**) Positive DAB staining in sections along the distal nephron for CaSR, TRPV5, TRPV6, SERCA1, SERCA2, PMCA1, PMCA4 and pPMCA. (**I**) As NCX2 is not expressed in the kidney, somata of trigeminal ganglion neurons served as a positive control for NCX2 staining. Scale bar: 20 μm. (PNG 70669 kb)

High resolution image (TIFF 1758 kb)

## Abbreviations

Ca^2+^: (Ionized) calcium
[Ca^2+^]_endolymph_: Endolymphatic Ca^2+^ concentration
Calb: Calbindin D-28k
CaSR: Calcium sensing receptor
CCD: Cortical collecting duct
ES: Endolymphatic sac
ED: Endolymphatic duct
MRC: Mitochondria-rich cell
NCX1/2: Sodium-calcium exchanger type 1/2
Parv: Parvalbumin
PMCA1/4: Plasma membrane calcium ATPase isoform 1/4
pPMCA: Pan-plasma membrane calcium ATPase
RRC: Ribosome-rich cell
SERCA1/2: Sarco/endoplasmic reticulum Ca^2+^-ATPase paralog 1/2
TRPV5/6: Transient receptor potential cation channel subfamily V member 5/6
V-ATPase: Vacuolar-type H^+^ ATPase
